# Association of reduced cerebrospinal fluid NPTX2 levels with postoperative delirium in patients undergoing knee/hip replacement: a prospective cohort study

**DOI:** 10.1007/s40520-023-02670-x

**Published:** 2024-02-17

**Authors:** Zongxiao Guo, Xiaoli Hong, Xiang Wang, Weiguo Chen, Zongfeng Guo

**Affiliations:** 1 Department of Orthopedic Surgery, Hai’an People’s Hospital, Haian, China; 2 Department of Anesthesiology, Hai’an People’s Hospital, Haian, China

**Keywords:** Postoperative delirium, Neuronal pentraxin 2, Biomarkers, Synaptic activity

## Abstract

**Background:**

Postoperative delirium (POD) is a common complication with poor prognosis in the elderly, but its mechanism has not been fully elucidated. There is evidence that the changes in synaptic activity in the brain are closely related to the occurrence of POD. And neuronal pentraxin 2 (NPTX2) can regulate synaptic activity in vivo.

**Aims:**

This study aims to explore whether decreased NPTX2 levels affects POD and whether the cerebrospinal fluid (CSF) biomarkers of POD mediate this association.

**Methods:**

In this prospective cohort study, we interviewed patients with knee/hip replacement 1 day before surgery to collect patient information and assess their cognitive function. CSF was extracted for measuring the CSF levels of NPTX2 and other POD biomarkers on the day of surgery. And postoperative follow-up visits were performed 1–7 days after surgery.

**Results:**

Finally, 560 patients were included in the study. The patients were divided into POD group and NPOD (non-POD) group. The POD group had a median age of 80 years, a female proportion of 45%, a median BMI of 24.1 kg/m^2^, and a median years of education of 9 years. The Mann–Whitney U test showed that CSF NPTX2 levels were significantly lower in POD group, compared with the NPOD group (*P* < 0.05). Univariate binary logistic regression analysis showed that reduced CSF levels of NPTX2 protected against POD (crude OR = 0.994, 95% CI 0.993–0.995, *P* < 0.001). The receiver-operating characteristic (ROC) curve indicated that CSF NPTX2 level had high predictive value for POD. Mediation analyses showed that CSF T-tau (mediating proportion = 21%) and P-tau (mediating proportion = 29%) had significant mediating effects on the association between CSF NPTX2 and POD.

**Conclusion:**

CSF NPTX2 levels were associated with the occurrence of POD. Low CSF NPTX2 levels may be an independent protective factor for POD. CSF T-tau and P-tau could mediate the association between CSF NPTX2 and POD occurrence.

**Clinical trial registration:**

The trial registration number (TRN): ChiCTR2200064740, Date of Registration: 2022-10-15.

**Supplementary Information:**

The online version contains supplementary material available at 10.1007/s40520-023-02670-x.

## Introduction

Postoperative delirium (POD) is defined as acute impairments in attention, awareness, and cognition [1]. POD is more common in the elderly, and thus, it poses a great challenge to our society with a rapidly aging population. POD usually brings about serious adverse effects on patients’ function and quality of life, extensive social impacts, as well as substantial health care costs [2]. Recent studies on POD focused on its preoperative, intraoperative, and postoperative risk factors [3]. Since POD-related biomarkers in cerebrospinal fluid (CSF) or plasma before operation have been reported to predict the occurrence of POD [4–6], early preoperative intervention could be taken to reduce the incidence of POD.

Although the specific mechanism of POD is still unclear, but there is growing evidence that changes in synaptic activity in the brain are closely related to the occurrence of POD [7]. Synaptic plasticity in the hippocampus directly affects hippocampal formation and function, and thus, it provides the biological basis for hippocampus-dependent learning, memory, and cognition. The dysfunction and loss of synapses may be one of the earliest pathological processes in neurodegeneration [8]. Biomarkers of synaptic activity may be useful for early diagnosis and monitoring of POD in clinical practice and clinical trials.

Neuronal pentraxins (NPTXs) are a family of three proteins [neuronal pentraxin 1 (NPTX1), neuronal pentraxin 2 (NPTX2), and neuronal pentraxin receptor (NPTXR)], which belong to the pentraxin family proteins (PTX) [9]. NPTXs are involved in homeostatic synaptic function and plasticity by recruiting postsynaptic receptors into the synapse. NPTX1 and NPTX2 play opposite regulatory roles in synaptic plasticity. NPTX1 limits excitatory synaptic plasticity and reduces the number of excitatory synaptic neurons [10]. And its exocrine secretion may promote hypoxic–ischemic neuronal death possibly through surface clustering with GluR1 at synaptic sites [11]. In addition, it has been demonstrated that β-amyloid (Aβ)-associated synaptic loss, ganglion damage, and neuronal death are mediated by increased NPTX1 expression [12]. In contrast, NPTX2 is activated in response to high neuronal activity and it promotes synaptic plasticity of excitatory synapses [13]. It regulates complement activity and inhibits microglia-mediated synaptic loss in neurodegeneration [9]. NPTX2 also has a bidirectional relationship with brain-derived neurotrophic factor (BDNF) [14]. In recent years, NPTX2 has been reported to be a promising synapse-derived biomarker for the progression of genetic frontotemporal dementia [15]. A decrease in NPTX2 was observed in the CSF of Alzheimer’s disease (AD) patients and NPTX2 showed robust correlations with cognitive function and hippocampal volume [16]. The NPTXR protein is a member of the neuronal pentapeptide family and is mainly expressed in the brain, with the highest expression observed in the hippocampus and cerebellum [17]. This transmembrane presynaptic protein is thought to be involved in the activation of excitatory and inhibitory neurons [18]. Recent studies have shown that NPTXR is a novel biomarker for the progression of neurodegenerative diseases such as AD, and CSF NPTXR levels decrease with the severity of AD [19, 20].

The aim of this study was to investigate whether the decrease of NPTX2 affected POD and the mediating role of CSF biomarkers in this association.

## Methods

### Included participants

This study included Han patients who received combined spinal and epidural anesthesia in Haian People’s Hospital from November 2022 to July 2023. All the patients before being included in this study have provided informed consent. Participants who simultaneously met the following inclusion criteria were included: (1) age ≥ 65; (2) ASAI-III; (3) body weight 50–90 kg; and (4) adequate education level to complete preoperative cognitive function tests. The exclusion criteria include: (1) central nervous system infection, head trauma, multiple sclerosis, epilepsy, or other major neurological disorders; (2) major psychological disorders (such as depression, delirium, etc.); (3) a preoperative Mini-Mental State Examination (MMSE) score < 23 points; (4) recent major surgery; (5) severe visual and hearing impairments; (6) family history of genetic diseases (7) serious systemic diseases (e.g., malignancies) that may affect the CSF level of POD biomarkers; (8) long-term use of psychotropic drugs, steroid drugs, and hormones; (9) abnormal preoperative coagulation function.

**Fig. 1:**
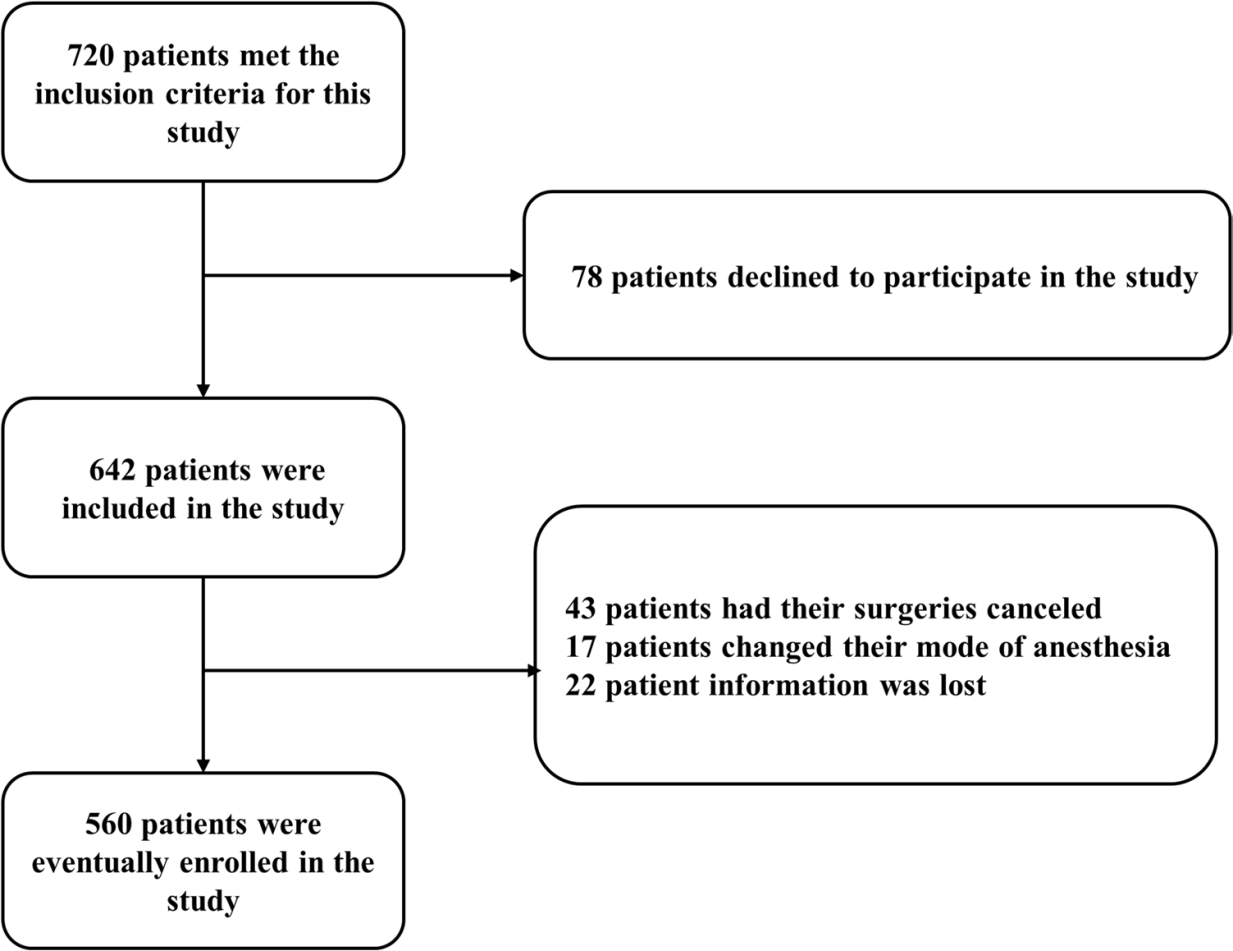
Flow diagram

A total of 560 cognitively normal participants had available information on covariates. They were divided into POD group and no postoperative delirium (NPOD) group. A patient recruitment flowchart is shown in Fig. 1.

### Preoperative evaluation

We interviewed all the patients the day before surgery and collected their baseline data, including age, gender, body mass index (BMI), years of education, and history of smoking and drinking. Other information, including histories of hypertension, diabetes mellitus, coronary heart disease (CHD), and cerebrovascular accident (CVA), was collected according to the patients’ medical records. All the history collection, physical examination, and dementia-related cognitive assessments were performed by an anesthesiologist.

### Anesthesia and surgery

The participants were told not to eat for 8 h and not to drink for 6 h before surgery, and they were not given any preoperative drugs. As soon as the patient entered the operation room, the venous access was opened; 3 mL of whole venous blood was drawn; and the ECG, SpO_2_, and NBP were regularly checked. All patients received combined spinal and epidural anesthesia. The puncture location was the lumbar spinous process region L3–4. Following a successful puncture, 2 mL of CSF fluid was removed from the subarachnoid area before spinal anesthesia, and 2–2.5 mL of levobupivacaine (0.66%) were then injected for roughly 30 s. Furthermore, we did not perform a second lumbar puncture. Following anesthesia, the sensory level was kept under T8. To keep the blood pressure within ± 20% of the baseline value during the procedure, oxygen was administered through mask at a rate of 5 L/min. Ephedrine 5 mg was administered intravenously if intraoperative NBP was below 90 mmHg (1 mmHg = 0.133 kPa) or had dropped by more than 20% from the baseline value. The patient was taken to the post-anesthesia care unit (PACU), or post-anesthesia care unit, after the procedure.

### Measurements of CSF samples

CSF samples were processed immediately within 2 h after standard lumbar puncture. Each sample was centrifuged at 2000 × g for 10 min, and CSF samples were separated and stored in an enzyme-free EP (Eppendorf) tube (AXYGEN; PCR-02-C) at − 80 °C under the international BIOMARKAPD project for further use in the subsequent steps of this study.

CSF biomarkers of POD were measured by enzyme linked immunosorbent assay (ELISA) using the microplate reader (Thermo Scientific Multiskan MK3). CSF biomarkers of POD measurements were done with other ELISA kits [Aβ42 (BioVendor, Ghent, Belgium Lot: No.296-64401), phosphorylated tau (P-tau) (BioVendor, Ghent, Belgium Lot: QY-PF9092), total tau (T-tau) (BioVendor, Ghent, Belgium Lot: No. EK-H12242), and NPTX2 (MEIMIAN, China, Lot: MM-51089H2)]. All ELISA measurements were performed by experienced technicians in strict accordance with the manufacturer’s instructions. The clinical data were hidden from them. The samples and standards were measured in duplicate, and the means of duplicates were used for the statistical analyses. To eliminate batch variation, all the antibodies and plates were from the same lot. Moreover, the within-batch CV (coefficient of variation) was < 5% and the inter-batch CV was < 15%.

#### Cognitive assessments

The preoperative cognitive status was assessed by neurologists using the MMSE [21]. Patients with an MMSE score < 23 points were excluded.

The POD assessment was performed at 9:00–10:00 am and at 2:00–3:00 pm twice a day on 1–7 days (or before discharge) by an anesthesiologist postoperatively. We used the visual analog scale (VAS) score of 0–10 (lower scores indicating lower levels of pain) to assess pain at the same time. POD was defined by the Confusion Assessment Method (CAM) [22], and POD severity was measured using the Memorial Delirium Assessment Scale (MDAS) [23]. Therefore, the postoperative conditions of CAM-positive and MDAS-positive patients were recorded.

#### Statistical analysis

##### Sample size calculation

The preliminary test in this study found that nine covariates were expected to enter the Logistic regression. The POD incidence was 15% [24]. And the loss of follow-up rate was assumed to be 20%. Therefore, the required sample size was calculated to be 720 (9 × 10 ÷ 0.15 × 1.2 = 720) [25].

##### Outcome analysis

For continuous variables, the demographic parameters were summed up as median (Q1, Q3), and for categorical categories, as number (column percentage). *P* values are used to compare between-group differences. The measurement data were examined to see if they complied with the normal distribution using the Kolmogorov–Smirnov test, either the Mann–Whitney U test (skewed distribution) or the *t* test (normally distributed data) for continuous variables. × 2 tests are used with category variables.

First, we used an unadjusted logistic regression (Model 1) to analyze the association between NPTX2 and POD. Univariate binary logistic regression analyses were carried out separately for the associations of POD with NPTX2, Aβ42, T-tau, P-tau, Aβ42/T-tau, and Aβ42/P-tau.

Second, we built two models to explore the robustness of the main results. Model 2 had additional adjustment for age (continuous variable), BMI (continuous variable), years of education (continuous variable), and MMSE scores (continuous variable). Model 3 is our sensitivity analysis by limiting the participants to over 70 years old on the basis of Model 2. Multivariate logistic regression analysis were used.

Thirdly, after applying multivariate Logistic regression to analyze the influencing factors of POD, the predicted value of POD by NPTX2 was analyzed by drawing the ROC curve and calculating the AUC value.

Finally, to examine whether the association between NPTX2 and POD was mediated by CSF POD biomarkers, logistic regression models were fitted based on the methods. The first equation regressed the mediator (CSF POD biomarkers) on the independent variable (NPTX2). The second equation regressed the dependent variable (POD) on the independent variable (NPTX2). The third equation regressed the dependent variable on both the independent variable and the mediator variable. Mediation effects were established if the following criteria were simultaneously reached: (1) NPTX2 were significantly related to CSF POD biomarkers; (2) NPTX2 were significantly related to POD; (3) CSF POD biomarkers were significantly related to POD; and (4) the association between NPTX2 and POD was attenuated when CSF POD biomarkers (the mediator) were added in the regression model. Furthermore, the attenuation or indirect effect was estimated, with the significance determined using 10,000 bootstrapped iterations, where each path of the model was controlled for age, gender, years of education, and MMSE scores.

The data were analyzed using SPSS version 23.0 (SPSS, Inc, Chicago, Illinois, USA), R software version 4.4.1(R Foundation for Statistical Computing, Vienna, Austria), and Stata MP16.0 (Solvusoft Corporation, Inc, Chicago, Illinois, USA). A *P* value < 0.05 was considered significant except where specifically noted.

## Results

### Participant characteristics

We initially recruited 720 participants. After excluding 160 participants (the reasons for dropping out are shown in Fig. 1), the remaining 560 were finally included in our study. Of the 560 included patients, 112 subjects experienced POD within 7 days after operation or before discharge. The incidence of POD was 20%. Participants were divided into two groups based on the presence or absence of POD (POD group and NPOD group). The demographic and clinical data of the two groups are summarized in Table 1.

**Table 1:** Characteristics of included participants

Characteristics	All participants (*n* = 560)		*P* value^*^
	POD (*n* = 112)	NPOD (*n* = 448)	
Age, year (median, Q1, Q3)	80 (69, 86.75)	69 (67, 75)	0.000^***^
Female, yes (%)	50 (45%)	243 (54%)	0.073
BMI, kg/m^2^	24.1 (20.0, 29.3)	26.5 (22.6, 29.00)	0.035^*^
Education, year (median, Q1, Q3)	9 (6–9)	9 (6–9)	0.001^**^
Hypertension, yes (%)	59 (53%)	248 (55%)	0.671
Diabetes mellitus, yes (%)	35 (31%)	165 (37%)	0.321
CHD, yes (%)	57 (51%)	221 (49%)	0.833
CVA, yes (%)	56 (50%)	214 (48%)	0.674
Smoking history, yes (%)	46 (41%)	187 (42%)	0.915
Drinking history, yes (%)	47(42%)	169 (38%)	0.448
MDAS, score (median, Q1, Q3)	17 (14, 27)	6 (3, 6)	0.000^***^
MMSE, score (median, Q1, Q3)	28 (27, 29)	28 (27, 29)	0.005^**^
CSF biomarkers (pg/mL)			
Aβ42	281.1 (187.6, 446.7)	336.99 (198.73, 475.42)	0.008^**^
T-tau	402.9 (234.0, 622.4)	233.39 (165.33, 313.18)	0.000^***^
P-tau	68.39 (45.5, 89.3)	40.30 (27.01, 49.29)	0.000^***^
Aβ42/T-tau	0.73 (0.51, 1.10)	1.5 (0.9, 2.3)	0.000^***^
Aβ42/P-tau	4.47 (2.78, 6.92)	8.8 (5.2, 13.2)	0.000^***^
NPTX2	424.8 (338.1, 687.3)	1126.95 (815.29, 1457.95)	0.000^***^

*CSF* cerebrospinal fluid, *NPTX2* neuronal pentraxins 2, *Aβ* amyloid-β, *T-tau* total tau, *P-tau* phosphorylated tau, *CHD* coronary heart disease, *CVA* cerebral vascular accident; data are presented as median (IQR) unless otherwise indicated

^*^P value < 0.05

^**^P value < 0.01

^***^P value < 0.001

### Correlation analysis of NPTX2 and CSF POD biomarkers

**Fig. 2:**
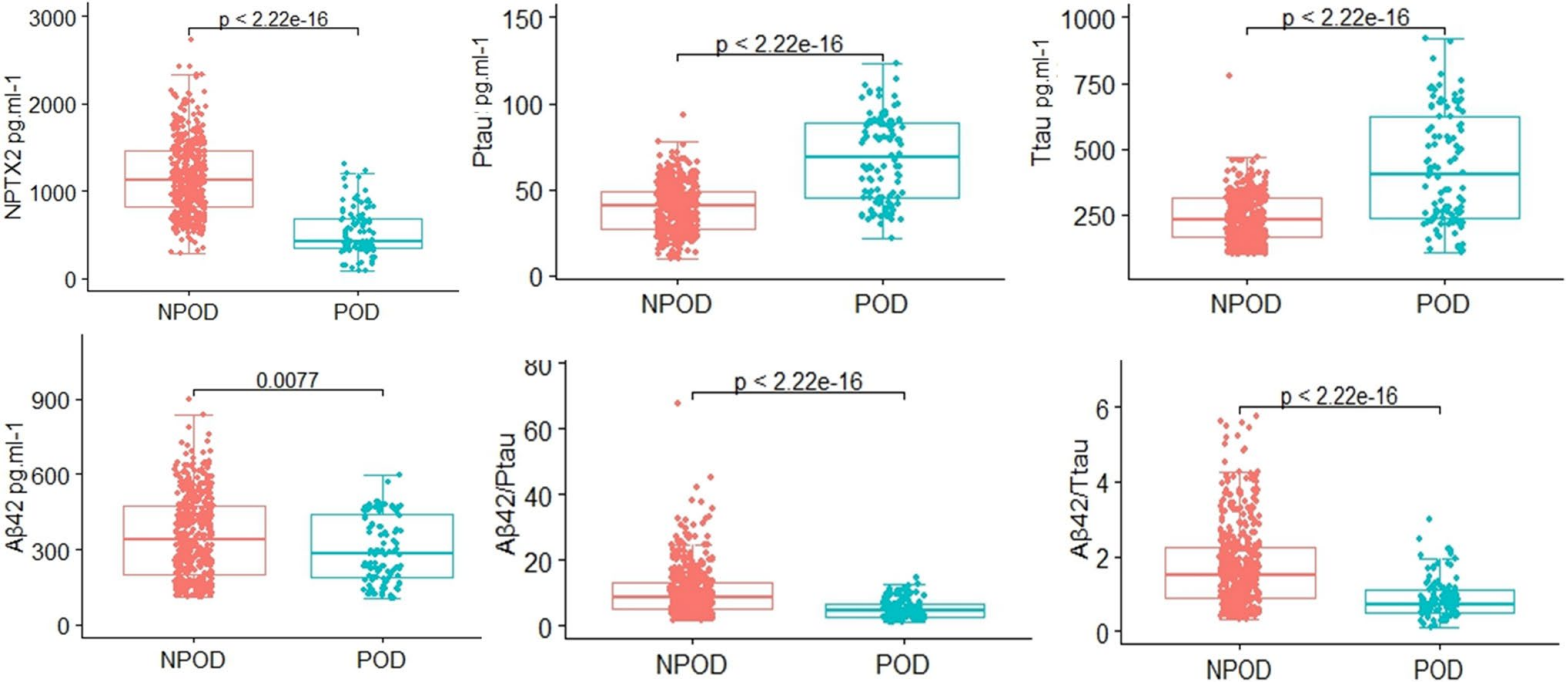
The box-plots. The box-plots showed the level of NPTX2 and CSF POD biomarkers in the POD group and the NPOD group. The result showed that CSF P-tau and T-tau levels of the participants were higher in the POD group. *NPTX2* neuronal pentraxins 2, *Aβ* amyloid-β, *T-tau* total tau, *P-tau* phosphorylated tau

The Mann–Whitney U test showed that NPTX2 and CSF biomarkers (Aβ42, T-tau, P-tau, Aβ42/T-tau, and Aβ42/P-tau) were associated with POD (*P* < 0.05) (Table 1). Patients with POD had significantly lower CSF levels of NPTX2, Aβ42, Aβ42/T-tau, and Aβ42/P-tau but significantly higher CSF levels of T-tau and P-tau than NPOD group (Fig. 2).

Univariate binary logistic regression analysis showed that the CSF levels of T-tau (crude OR = 1.009, 95% CI 1.007–1.011, *P* < 0.001) and P-tau (crude OR = 1.085, 95% CI 1.068–1.102, *P* < 0.001) were risk factors for POD, while the CSF levels of NPTX2 (crude OR = 0.994, 95% CI 0.993–0.995, *P* < 0.001), Aβ42 (OR = 0.998, 95%CI 0.996–0.999, *P* = 0.003), Aβ42/T-tau (crude OR = 0.252, 95% CI 0.171–0.372, *P* < 0.001), and Aβ42/P-tau (crude OR = 0.770, 95% CI 0.716–0.827, *P* < 0.001) were protective factors of POD.

After adjustment for age, BMI, years of education, and MMSE score, the above associations remained significant. CSF T-tau (crude OR = 1.010, 95% CI 1.008–1.013, *P* < 0.001) and P-tau (crude OR = 1.094, 95% CI 1.073–1.116, *P* < 0.001) remained risk factors for POD. CSF NPTX2 (crude OR = 0.994, 95% CI 0.993–0.996, *P* < 0.001), Aβ42 (OR = 0.998, 95%CI 0.996–0.999, *P* < 0.001), Aβ42/T-tau (crude OR = 0.225, 95% CI 0.142–0.356, *P* < 0.001), and Aβ42/P-tau (crude OR = 0.758, 95% CI 0.698–0.824, *P* < 0.001) were still protective factors for POD.

Furthermore, sensitivity analyses were performed to verify the robustness of our results. We analyzed whether the associations would change if only individuals aged over 70 at the baseline were included. Our sensitivity analyses showed that the associations for NPTX2 (crude OR = 0.995, 95% CI 0.993–0.997, *P* < 0.001) and CSF POD biomarkers remained stable (Table 2).

**Table 2:** Logistic regression analysis and sensitivity analysis

	Model 1		Model 2		Model 3	
	OR(95%CI)	*P* value	OR(95%CI)	*P* value	OR(95%CI)	*P* value
NPTX2 (pg/mL)	0.994(0.993–0.995)	< 0.001^***^	0.994(0.993–0.996)	< 0.001^***^	0.995(0.993–0.997)	< 0.001^***^
Aβ42 (pg/mL)	0.998(0.996–0.999)	0.003^**^	0.998(0.996–0.999)	< 0.001^***^	0.997(0.995–1.000)	< 0.001^***^
T-tau (pg/mL)	1.009(1.007–1.011)	< 0.001^***^	1.010(1.008–1.013)	< 0.001^***^	1.012(1.009–1.016)	< 0.001^***^
P-tau (pg/mL)	1.085(1.068–1.102)	< 0.001^***^	1.094(1.073–1.116)	< 0.001^***^	1.098(1.068–1.130)	< 0.001^***^
Aβ42/T-tau	0.252(0.171–0.372)	< 0.001^***^	0.225(0.142–0.357)	< 0.001^***^	0.202(0.106–0.386)	< 0.001^***^
Aβ42/P-tau	0.770(0.716–0.827)	< 0.001^***^	0.758(0.698–0.824)	< 0.001^***^	0.778(0.701–0.864)	< 0.001^***^

*NPTX2* neuronal pentraxins 2, *Aβ* amyloid-β, *T-tau* total tau, *P-tau* phosphorylated tau

Model 1: The unadjusted logistic regression

Model 2: Adjusted logistic regression, the adjustment factors include: Age, BMI, years of education, and MMSE

Model 3: Sensitivity analysis was based on selecting only individuals older then 70

^*^P value < 0.05

^**^P value < 0.01

^***^P value < 0.001

### Predictive model

**Fig. 3:**
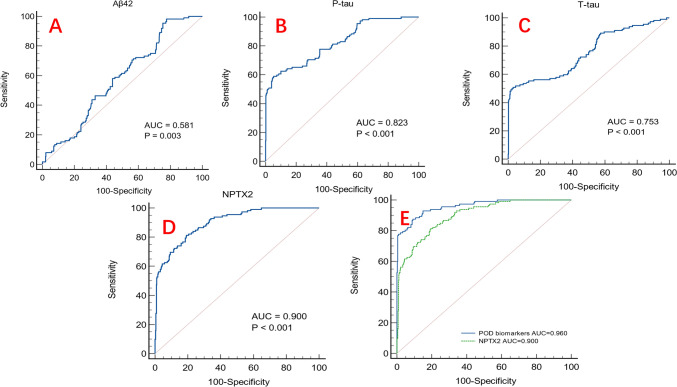
The ROC curve. The ROC curve showed that NPTX2, CSF POD biomarkers and the model combining NPTX2 and the CSF POD biomarkers had effective diagnostic significance in predicting POD occurrence. **A** The receiver-operator characteristic analyses for Aβ42 in predicting delirium. **B** The receiver-operator characteristic analyses for P-tau in predicting delirium. **C** The receiver-operator characteristic analyses for T-tau in predicting delirium. **D** The receiver-operator characteristic analyses for NPTX2 in predicting delirium. **E** The receiver-operator characteristic analyses for the model combining NPTX2 and the CSF POD biomarkers in predicting delirium. *NPTX2* neuronal pentraxins 2, *Aβ* amyloid-β, *T-tau* total tau, *P-tau* phosphorylated tau

Receiver-operating characteristics (ROC) curve showed that NPTX2, P-tau, and T-tau all have good value for predicting POD occurrence. NPTX2 had a satisfactory predictive power for POD with an area under the curve (AUC) of 0.900 (95% confidence interval [CI]: 0.872–0.923, *P* < 0.001). The model combining NPTX2 and POD-related biomarkers (AUC = 0.900; *P* < 0.001) also exhibited a relative discriminatory ability in POD prediction. The efficacy of each predictor is shown in the nomogram (Fig. 3).

### Causal mediation analyses

**Fig. 4:**
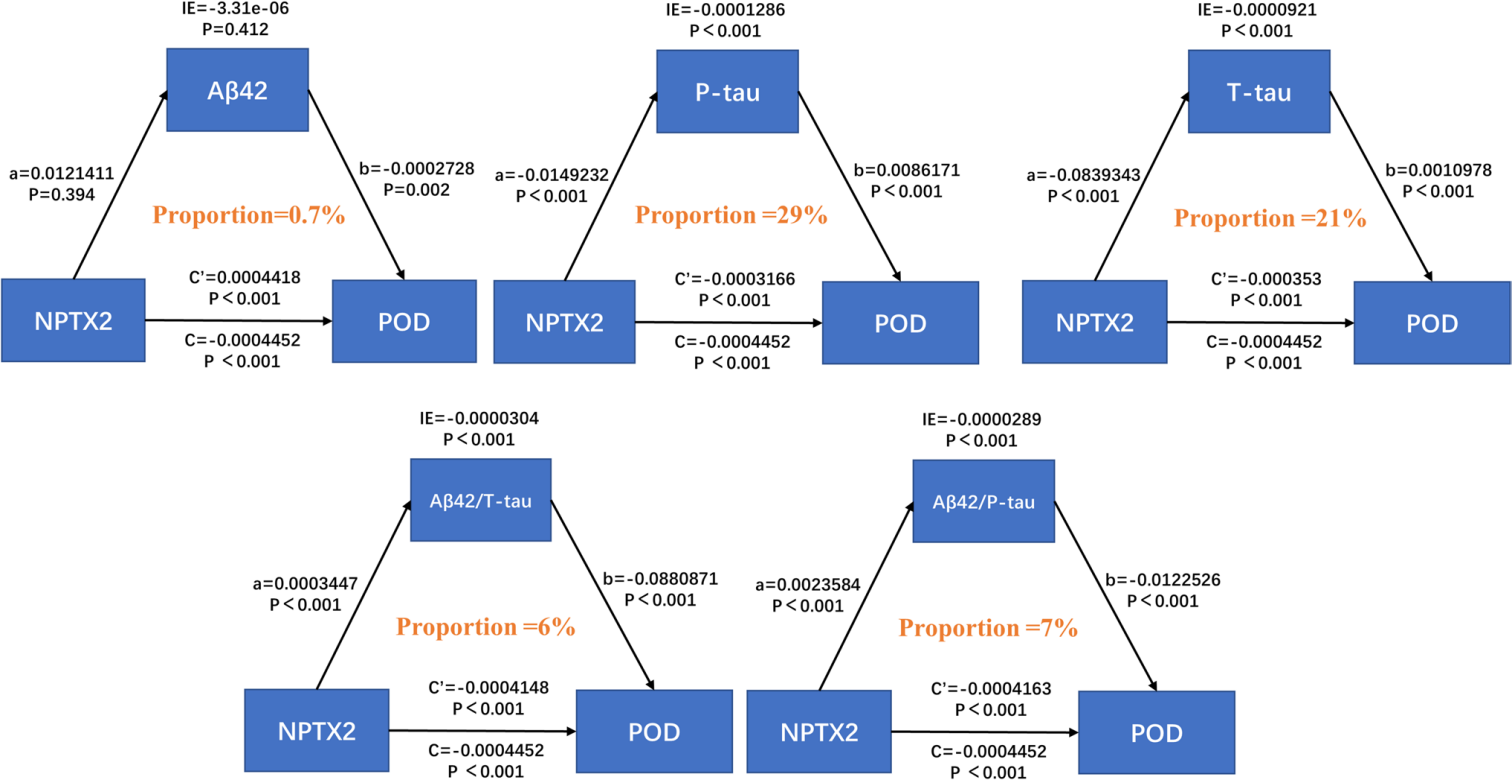
Mediation analyses; a represents the effect of the independent variable (NPTX2) on the mediator (Aβ42, T-tau, P-tau, Aβ42/T-tau, Aβ42/P-tau), b represents the effect of the mediator (Aβ42, T-tau, P-tau, Aβ42/T-tau, Aβ42/P-tau) on the dependent variable (POD), *c* represents total effect, *c*′ represents direct effect, IE represents indirect effect, The mediating effect (proportion) is obtained by dividing the indirect effect by the total effect. The mediation analysis showed that the relationship between NPTX2 and POD was mediated by T-tau and P-tau protein. *NPTX2* neuronal pentraxins 2, *Aβ* amyloid-β, *T-tau* total tau, *P-tau* phosphorylated tau

To explore whether NPTX2 affects the occurrence of POD through CSF POD biomarkers, we conducted causal mediation analyses. The mediation analyses showed that the relationship between NPTX2 and POD was mediated by T-tau (mediating proportion = 21%) and P-tau (mediating proportion = 29%) (Fig. 4). Among these CSF POD biomarkers, P-tau had the largest mediating effect on the association between NPTX2 and the occurrence of POD.

## Discussion

Our study found that NPTX2 was associated with the occurrence of POD, and low CSF levels of NPTX2 might be an independent predictor of POD. POD is characterized by acute cognitive dysfunction after operation, and it is closely related to changes in synaptic activity in the brain [7]. However, complement in the brain can mediate synaptic elimination in a variety of nervous system diseases. Studies have shown that NPTX2 can improve microglia-mediated synaptic loss by inhibiting complement activity in the brain, and the decrease of NPTX2 concentration may exacerbate complement-mediated neurodegeneration [9]. In recent years, NPTX2 has been suggested to provide additional and independent prediction of the onset of mild cognitive impairment (MCI) in cognitively normal individuals, and it might serve as an important prognostic biomarker during preclinical AD [26]. In addition to CSF NPTX2 levels, serum NPTX2 levels are also thought to be independently associated with cognitive function and can be used as a predictor of cognitive function [27]. Anesthesia and surgery selectively induce a functional reduction in excitatory synaptic transmission of PFC in the prefrontal cortex [28], thus inducing the occurrence of POD. As POD is a common perioperative complication in elderly patients, it will affect the length of hospital stay and long-term prognosis of patients. Therefore, accurate identification of patients at high risk for POD and timely intervention for them before surgery can reduce the incidence of POD, shorten the length of hospital stay, and improve their long-term prognosis.

This study indicated that NPTX2 was an independent protective factor for POD, and the incidence of POD decreased with the increase of CSF NPTX2 level. After additional adjustment for age, gender, years of education, and MMSE scores, the Model 2 showed the same results. What’s more, the sensitivity analysis by only including those over 70 years showed that the result did not change, indicating that the conclusion that NPTX2 is a protective factor for POD occurrence is robust.

In addition, ROC curve analysis in this study showed that NPTX2 had a satisfactory predictive ability for POD, and the combination of NPTX2 and POD-related CSF biomarkers is a good predictor of POD under the same model.

To explore the relationship between NPTX2 and the pathogenesis of POD, our study investigated the intermediary role of CSF biomarkers in the association between NPTX2 and POD and found that T-tau and P-tau proteins accounted for the majority of the mediating effects. Previous studies showed that NPTX2 acted on the long-term enhancement of synaptic plasticity and synaptic strength [13, 29, 30]. Tau proteins have been shown to be involved in normal synaptic transmission and normal neuronal function under physiological conditions [31]. They can regulate the synaptic plasticity of hippocampal neurons in response to brain-derived neurotrophic factor [32]. And tau proteins are also involved in synaptic dysfunction and memory impairment [33]. For example, they regulated vesicle release and presynaptic function by directly interacting with a vesicle protein called Synaptogrin-3 [34]. And the abnormal post-translational modifications of tau proteins might trigger and worsen synaptic dysfunction [35]. In this study, we revealed that NPTX2 can influence POD occurrence via the mediating effect of tau proteins. The underlying mechanism may be their regulation (NPTX2 and tau proteins) of synaptic function. In the future, preoperative CSF NPTX2 level can be measured to identify the high-risk population for POD, and then, timely interventions will be taken for them to reduce the incidence of POD.

This study still has several limitations. First, as a cross-sectional study, our study did not monitor the dynamic changes in CSF NPTX2 levels and did not have cognitive outcomes during long-term follow-up. In the future, more cohort studies are needed to explore the longitudinal correlation between NPTX2 and POD. In addition, the study only included patients over the age of 65, and the generalizability of our results may be limited. In the future, it is necessary to conduct large-scale multi-center studies across different age groups. Finally, this study excluded patients with a preoperative MMSE score < 23 and depressed patients. Therefore, the relationship between NPTX2 and POD in these patients needs to be further explored.

## Conclusion

In conclusion, our study showed that NPTX2 was related to the occurrence of POD and low CSF NPTX2 levels might be an independent predictor of POD. In addition, the relationship between NPTX2 and POD occurrence was mediated by T-tau and P-tau proteins.

### Supplementary Information

Below is the link to the electronic supplementary material.

Supplementary file1 (XLSX 77 KB)

## Data Availability

The datasets used and/or analyzed in this study may be provided at the reasonable request of the corresponding author.
